# Solvent-Free and Microwave-Assisted Synthesis Enables Formation of Imidazole and Pyrazole Derivatives Through Epoxide Ring Opening

**DOI:** 10.3390/molecules30081760

**Published:** 2025-04-14

**Authors:** MaryGrace McAfee, Joshua Pack, Brian Walker

**Affiliations:** Department of Chemistry, University of Arkansas at Little Rock, 2801 S. University Ave Science Lab Building 451, Little Rock, AR 72204, USA; memcafee1@ualr.edu (M.M.); jlpack@ualr.edu (J.P.)

**Keywords:** microwave synthesis, solvent free, imidazoles, azoles, antifungal

## Abstract

A solvent-free, microwave-assisted approach for the ring-opening reactions of phenyl glycidyl ether with a series of commercially available imidazoles and pyrazoles is described. Microwave irradiation allows reactions to proceed rapidly. This straightforward approach efficiently generated adducts with competitive yields compared to traditional methods that use conventional heating or organic solvents. This technique is particularly suited for high-throughput screening in drug discovery, offering a significant reduction in time and resource consumption.

## 1. Introduction

Solvent-free and microwave-assisted synthesis techniques are important in modern chemistry for several reasons. First, solvent-free methods are a sustainable and environmentally friendly approach to chemical synthesis [1,2,3,4,5]. Additionally, microwave-assisted synthesis techniques represent an advancement in sustainability and offer considerable benefits in terms of environmental responsibility, speed, and selectivity [6,7,8,9,10,11,12,13,14]. Rapidly heating reaction mixtures through microwave irradiation can often complete a synthesis in a fraction of the time required by conventional methods [4,6,7,8,9,10,11,12,13,14]. Considering these advantages, we explored this approach in the development of substituted azole compounds. Azoles are an important class of organic compounds characterized by a five-membered ring containing at least one nitrogen atom [15]. These compounds have shown significant utility in medicinal chemistry due to their biological activities [16,17,18].

**Figure 1:**
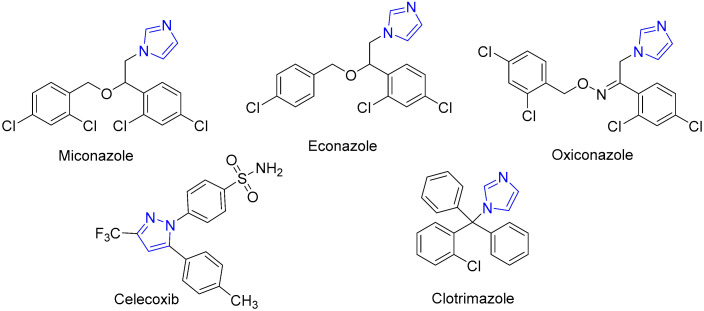
Selected examples of medicinally relevant azole-containing compounds.

Celecoxib, for example, sold under the brand name Celebrex^®^ (Pfizer, New York, NY, USA), contains a pyrazole ring and is a nonsteroidal anti-inflammatory drug (NSAID) used for its analgesic and anti-inflammatory properties [19] (Figure 1). Celecoxib selectively targets cyclooxygenase-2 (COX-2) enzymes, reducing pain and inflammation without affecting the COX-1 enzymes responsible for protecting the stomach lining [19]. Imidazoles are another important example of an azole ring that plays a crucial role in many medicines [20].

For example, miconazole, econazole, oxiconazole, and clotrimazole are all widely used antifungal medications that contain an imidazole ring [20] (Figure 1). Miconazole, sold under the brand name Monistat^®^ (Prestige Consumer Healthcare, Lynchburg, VA, USA), works by inhibiting ergosterol, a crucial part of the fungus membrane, ultimately leading to the elimination of the infection [21].

However, due to emerging antifungal resistance to current medications [22], and more broadly the prevalence of azoles in natural and medicinal compounds, our group explored solvent-free and microwave-assisted synthesis techniques for the development of compounds derived from 1,2- and 1,3-substituted azoles. Herein, we describe a general and efficient protocol for solvent-free and microwave-assisted reactions of substituted pyrazoles and imidazoles with phenyl glycidyl ether as the electrophile. The nucleophilicity of the azole ring, paired with the reactivity of epoxide as electrophiles, allows for the formation of versatile derivatives that can be further enhanced for drug potency, specificity, and pharmacokinetic properties, enabling more effective therapies. These compounds’ and other azole compounds’ value can also be found in their versatility of pharmacological properties and mechanisms of action.

## 2. Results

Nucleophilic ring-opening reactions of phenyl glycidyl ether (**1**) with azoles have been adequately reported in previous work [23,24,25]. Some methods of approach have included the use of a Lewis acid/base catalyst, strong bases at high temperatures, and some solvent-free reaction conditions. One such example highlighted solvent-free conditions, reporting that traditional heating at 60 °C for 12 h in the absence of solvent resulted in an 82% yield of product (**3a**) [26] (Table 1, entry 1). Another example described solvent-free reaction conditions with Yb(OTf)_3_ as a Lewis acid catalyst and reportedly afforded (**3a**) in 80% yield [27] (Table 1, entry 2). However, in our case, using a milligram reaction scale, the solvent-free and conventional heating method and the Lewis acid-catalyzed conditions consistently afforded more modest yields: 55% and 47%, respectively. Due to the low yield and reproducibility problems in our best attempts, we decided to use microwave-assisted heating in the evaluation of our experiments.

Using imidazole as a nucleophile, we studied the effects of temperature, reaction time, and molar ratio of nucleophile to the epoxide, phenyl glycidyl ether. The experiments were performed using an Anton Paar Mono-wave 400 instrument equipped with an IR temperature sensor, and an internal camera was used to monitor the progress of the reactions. We started our investigation with a 1:1 mixture of phenyl glycidyl ether (**1**) and the imidazole (**2a**) placed in a pressure tube and heated in the microwave oven at 150 °C for 5 min (Table 1, entry 3). TLC showed multiple spots that had similar retention times, which made column chromatography of the dark amber mixture difficult. Shorter reaction times at 150 °C also showed several spots of the dark amber mixture, indicating decomposition at those higher temperatures. When reducing the microwave temperature to 60 °C, the mostly clear reaction mixture showed minimal formation of the product, and both starting materials remained after 5 min. Longer reaction times, up to ten minutes, seemed to have no effect on the outcome of the reaction when using temperatures below 80 °C in the microwave. Ultimately, heating to 120 °C for 1 min was found to provide the best yields (Table 1, entry 7), comparable to efforts made to reproduce the published procedures.

Although the yields did not exceed published literature values, the advantages of the microwave offered a quick and efficient synthetic pathway to a series of azole derivatives. and the yields were comparable when using the procedures described in the literature on an equivalent reaction scale. This methodology enhances reaction efficiency by minimizing processing time, making it a practical alternative to conventional approaches. To ensure the most efficient transformation into product **3a**, it was found that 1.5 equivalents of epoxide worked best. This excess of phenyl glycidyl ether ensured that imidazole **2a** was completely consumed during the rapid conversion in the microwave. The Rf of the epoxide was much different than the product, so consuming as much of the azole as possible made column purification easier. Furthermore, the reaction progress was easily monitored through the camera in the microwave, which showed that the phenyl glycidyl ether and imidazole *rapidly* combined into a viscous, light amber mixture. Slightly longer heating times (<2.5 min.) were not observed to have any effects on the reaction outcomes.

After establishing the reaction conditions for solvent-free microwave synthesis, we considered other commercially available derivatives of imidazole and pyrazole. Unless stated otherwise, we applied the same synthetic methodologies in creating the adduct list. Phenyl glycidyl ether (**1**) and the azole reacted together in a 1:1.5 ratio, heated solvent-free in the microwave to 120 °C for 1 min, and the reaction was subsequently monitored by TLC and purified by silica gel column chromatography. All the azoles reacted to give serviceable yields of the product, as displayed in Table 2. 

Admittedly, the goal in any synthetic endeavor is high yields; however, an overriding goal for this project was to develop a protocol for rapid, environmentally friendly, solvent-free, and microwave-assisted reactions of substituted azole compounds and phenyl glycidyl ether. Minimal steric repulsion was observed for the alkyl-substituted azole entries **3b**, **3c**, and **3f** in Table 2, with the lowest yield of 49%. Diminished yields were observed in **3d**, giving modest yields of 21%, and **3g** gave slightly higher yields of 26%. This was attributed to the large surface area halogens are known to occupy, and the electronic nature of the halogen attached to the nucleophilic rings.

## 3. Conclusions

The solvent-free and microwave-assisted conditions demonstrated in this work expand on the collection of synthetic techniques available for the ring opening of glycidyl ether with imidazole, pyrazole, and selected derivatives. The microwave’s unique ability to rapidly and consistently achieve reactive conditions necessary to afford products has potential to streamline small-molecule screening of azole derivatives with potential therapeutic activity. This efficiency is not achievable with traditional heating, with or without solvents, making this synthetic route favorable for small-compound screening.

## 4. Materials and Methods

### 4.1. Reagents and Equipment

All reagents were purchased from Millipore Sigma and used as received without further purification. Hexane, ethyl acetate, and acetone, used for purification, were of spectrophotometric grade. Silica gel (40–63 µm) and TLC silica gel 60 F254 were purchased from Sorbtech, East Norcross, GA, USA. Nuclear magnetic resonance spectra were measured on a JEOL (Pleasanton, CA, USA) NMR spectrometer operating at 400 MHz for ^1^H NMR and 100.6 MHz for ^13^C NMR. Free Induction Decays were processed on a Windows computer with the Delta 5.2.1 program (see Supplementary Materials). Chemical shifts are represented in ppm (s = singlet; d = doublet; t = triplet; m = multiplet) and referenced to a CDCl_3_ solvent peak unless stated otherwise.

### 4.2. General Method for Reactions of Phenyl Glycidyl Ether and Imidazole

To a dry microwave tube, an imidazole derivative and then phenyl glycidyl ether were added. The reaction mixture was heated to 120 °C over the course of 1 min by the microwave. The crude product was purified by flash chromatography.

#### 4.2.1. 1-(1*H*-imidazol-1-yl)-3-phenoxypropan-2-ol (3a)

To a dry microwave tube, imidazole (0.050 g, 0.733 mmol) and then phenyl glycidyl ether (0.165 g, 1.099 mmol) were added. The reaction mixture was heated to 120 °C over the course of 1 min by the microwave. The crude product was purified by flash chromatography and provided α-(Phenoxymethyl)-1H-imidazole-1-ethanol (0.0848 g, 53%) as off-white crystals. 1H-NMR (400 MHz, CHLOROFORM-D) δ 7.46 (s, 1H), 7.28–7.33 (m, 2H), 7.00 (t, J = 7.6 Hz, 1H), 6.93 (t, J = 8.0 Hz, 4H), 4.22–4.27 (m, 2H), 4.07–4.14 (m, 1H), 3.99 (q, J = 4.9 Hz, 1H), 3.92 (dd, J = 9.6 Hz, J = 6.0, 1H). 13C-NMR (101 MHz, CHLOROFORM-D) δ 158.18, 137.84, 129.74, 129.02, 121.61, 119.84, 114.57, 69.29, 68.72, 50.37.

#### 4.2.2. 1-(2-Methyl-1*H*-imidazol-1-yl)-3-phenoxypropan-2-ol (3b)

To a dry microwave tube, 2-methylimidazole (0.601 g, 0.732 mmol) and then phenyl glycidyl ether (0.165 g, 1.01 mmol) were added. The reaction mixture was heated to 120 °C over the course of 1 min by the microwave. The crude product was purified by flash chromatography and provided 2-Methyl-α-(phenoxymethyl)-1H-imidazole-1-ethanol (0.0824 g, 49%) as off-white crystals. 1H-NMR (400 MHz, CHLOROFORM-D) δ 7.31 (m, 2H), 7.00 (t, 7.3 Hz, 1H), 6.85–6.93 (m, 4H), 4.25 (m, 1H), 4.17 (dd, J = 14.2 Hz, J = 4.6 Hz, 1H), 4.02–4.05 (m, 1H), 3.97 (d, J = 5 Hz, 2H), 2.39 (s, 3H). 13C-NMR (101 MHz, CHLOROFORM-D) δ 158.14, 145.20, 129.57, 126.51, 121.31, 119.84, 114.40, 69.04, 68.80, 49.41, 12.94.

#### 4.2.3. 1-(2-Ethyl-4-methyl-1*H*-imidazol-1-yl)-3-phenoxypropan-2-ol (3c)

To a dry microwave tube, 2-ethyl-4-methylimidazole (0.0807 g, 0.733 mmol) and then phenyl glycidyl ether (0.165 g, 1.099 mmol) were added. The reaction mixture was heated to 120 °C over the course of 1 min by the microwave. The crude product was purified by flash chromatography and provided 2-Ethyl-5-methyl-α-(phenoxymethyl)-1H-imidazole-1-ethanol (0.0957 g, 50%) as a clear oil. 1H-NMR (400 MHz, CHLOROFORM-D) δ 7.31 (m, 2H), 7.00 (m, 1H), 6.91 (m, 2H), 6.59 (s, 1H), 4.20–4.24 (m, 1H), 4.10 (dd, J = 14.4 Hz, J = 4.8 Hz, 1H), 3.93–4.01 (m, 3H), 2.68 (q, J = 8.2 Hz, 2H), 2.16 (s, 3H), 1.27–1.31 (m, 3H). 13C-NMR (101 MHz, CHLOROFORM-D) δ 158.20, 149.35, 135.88, 129.73, 121.58, 115.98, 114.56, 69.51, 68.91, 48.68, 20.08, 13.48, 12.65.

#### 4.2.4. 1-(2-Iodo-1*H*-imidazol-1-yl)-3-phenoxypropan-2-ol (3d)

To a dry microwave tube, 2-iodoimidazole (0.142 g, 0.732 mmol) and then phenyl glycidyl ether (0.220 g, 1.465 mmol) were added. The reaction mixture was heated to 120 °C over the course of 1 min by the microwave. The crude product was purified by flash chromatography and provided 2-Iodo-α-(phenoxymethyl)-1H-imidazole-1-ethanol (0.0527 g, 21%) as a yellow oil. 1H-NMR (400 MHz, CHLOROFORM-D) δ 7.30 (t, J = 7.1 Hz, 2H), 7.18–7.22 (m, 1H), 6.98–7.12 (m, 2H), 6.91 (d, J = 7.8 Hz, 2H), 4.20–4.30 (m, 2H), 4.02–4.13 (m, 2H), 3.90 (q, J = 4.7 Hz, 1H), 2.77 (s, 0H). 13C-NMR (101 MHz, CHLOROFORM-D) δ 157.93, 132.73, 129.66, 124.26, 121.65, 114.48, 90.30, 77.31, 77.00, 76.68, 69.32, 68.57, 51.70.

#### 4.2.5. 1-Phenoxy-3-(1*H*-pyrazol-1-yl)propan-2-ol (3e)

To a dry microwave tube, pyrazole (0.050 g, 0.734 mmol) and then phenyl glycidyl ether (0.190 g, 1.099 mmol) were added. The reaction mixture was heated to 120 °C over the course of 1 min by the microwave. The crude product was purified by flash chromatography and provided 1-phenoxy-3-(1H-pyrazol-1-yl)propan-2-ol (0.117 g, 73%) as white crystals. 1H-NMR (400 MHz, CHLOROFORM-D) δ 7.53 (s, 1H), 7.42 (s, 1H), 7.24–7.29 (m, 2H), 6.87–6.98 (m, 3H), 6.25 (d, 1H), 4.62 (s, 1H), 4.30–4.46 (m, 3H), 3.94–3.97 (m, 1H), 3.73–3.77 (m, 1H). 13C-NMR (101 MHz, CHLOROFORM-D) δ 158.31, 140.03, 130.85, 129.66, 121.37, 114.55, 105.69, 69.46, 68.41, 53.93.

#### 4.2.6. 1-(3,5-Dimethyl-1*H*-pyrazol-1-yl)-3-phenoxypropan-2-ol (3f)

To a dry microwave tube, 3,5-dimethylpyrazole (0.0704 g, 0.732 mmol) and then phenyl glycidyl ether (0.165 g, 1.099 mmol) were added. The reaction mixture was heated to 120 °C over the course of 1 min by the microwave. The crude product was purified by flash chromatography and provided 3,5-dimethyl-α-(phenoxymethyl)-1H-pyrazole-1-ethanol (0.0913 g, 51%) as off-white crystals. 1H-NMR (400 MHz, CHLOROFORM-D) δ 7.25–7.29 (m, 2H), 6.87–6.98 (m, 3H), 5.79 (s, 1H), 4.80 (s, 1H), 4.16–4.33 (m, 3H), 4.01 (q, J = 4.6 Hz, 1H), 3.62 (t, J = 8.2 Hz, 1H), 2.21 (s, 3H), 2.18 (s, 3H). 13C-NMR (101 MHz, CHLOROFORM-D) δ 158.19, 148.20, 140.24, 129.55, 121.15, 114.38, 104.96, 69.35, 67.88, 49.29, 13.42, 10.81.

#### 4.2.7. 1-(3-Chloro-5-methyl-1*H*-pyrazol-1-yl)-3-phenoxypropan-2-ol (3g)

To a dry microwave tube, 3-chloro-5-methyl-1H-pyrazole (0.101 g, 1.054 mmol) and then phenyl glycidyl ether (0.190 g, 1.265 mmol) were added. The reaction mixture was heated to 120 °C over the course of 1 min by the microwave. The crude product was purified by flash chromatography and provided 3-Chloro-5-methyl-α-(phenoxymethyl)-1H-pyrazole-1-ethanol (.0619 g, 26%) as a yellow oil. 1H-NMR (400 MHz, CHLOROFORM-D) δ 7.28 (dd, J = 8.6 Hz, J = 7.4 Hz, 2H), 6.88–6.98 (m, 3H), 6.00 (s, 1H), 4.24–4.39 (m, 3H), 4.01–4.11 (m, 2H), 3.82 (dd, J = 9.6 Hz, J = 6.4 Hz, 1H), 2.22 (s, 3H). 13C-NMR (101 MHz, CHLOROFORM-D) δ 158.24, 149.27, 129.51, 128.16, 121.22, 114.49, 104.26, 69.25, 68.35, 50.04, 13.97.

## Figures and Tables

**Table 1:**
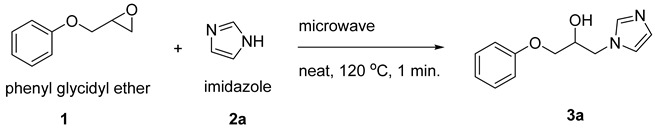
Reaction conditions for solvent-free phenyl glycidyl (**1**) ring opening with imidazole (**2a**).

Entry	Equiv. of Epoxide	Time (min.)	T (°C)	Yield (%)
1	1.0	720	60	56
2^a^	2.0	1440	25	47
3	1.0	5	150	trace
4	1.0	1	150	trace
5	1.0	5	60	<15
6	1.5	10	80	<15
7	1.5	1	120	53.0

^a^ used 5 mol% of Yb(Otf)_3_ as a catalyst.

**Table 2:**
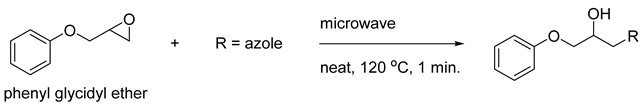
Conditions for solvent-free, microwave-assisted ring opening of phenyl glycidyl ether (**1**) with azoles (**2**).

Entry	Azole (2)	No.	Product	Yield (%)
1	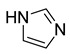	**3a**	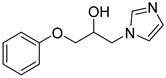	56
2	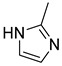	**3b**	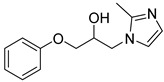	53
3	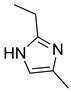	**3c**	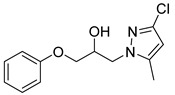	49
4	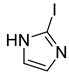	**3d**	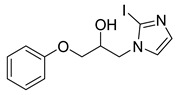	21
5	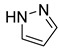	**3e**	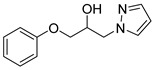	58
6	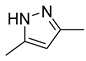	**3f**	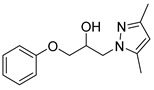	55
7	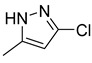	**3g**	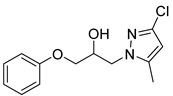	26

## Data Availability

The data presented in this study are contained within the article.

## Supplementary Materials

The following supporting information can be downloaded at: https://www.mdpi.com/article/10.3390/molecules30081760/s1. ^1^H and ^13^C NMR spectra of compounds 3a-3g were recorded in CDCl_3_ at 25 °C. Figure S1. 1H NMR spectra of compound 3a. Figure S2. 13C NMR spectra of compound 3a. Figure S3. 1H NMR spectra of compound 3b. Figure S4. 13C NMR spectra of compound 3b. Figure S5. 1H NMR spectra of compound 3c. Figure S6. 13C NMR spectra of compound 3c. Figure S7. 1H NMR spectra of compound 3d. Figure S8. 13C NMR spectra of compound 3d. Figure S9. 1H NMR spectra of compound 3e. Figure S10. 13C NMR spectra of compound 3e. Figure S11. 1H NMR spectra of compound 3f. Figure S12. 13C NMR spectra of compound 3f. Figure S13. 1H NMR spectra of compound 3g. Figure S14. 13C NMR spectra of compound 3g.

## Abbreviations

CDCl₃, deuterated chloroform; COX-1, cyclooxygenase-1; COX-2, cyclooxygenase-2; EtOAc, ethyl acetate; IR, infrared; MeOH, methanol; MW, microwave; NMR, nuclear magnetic resonance; NSAID, nonsteroidal anti-inflammatory drug; PGE, phenyl glycidyl ether; Rf, retention factor; THF, tetrahydrofuran; TLC, thin-layer chromatography; Yb(OTf)₃, ytterbium(III) trifluoromethanesulfonate.
