# Supplementary material for: Solvent-Free and Microwave-Assisted Synthesis Enables Formation of Imidazole and Pyrazole Derivatives Through Epoxide Ring Opening
Source: Molecules. 2025 Apr 14;30(8):1760. doi: 10.3390/molecules30081760 (PMC12029546; doi:10.3390/molecules30081760)

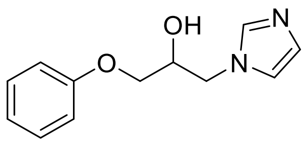

(3a) To a dry microwave tube was added imidazole (0.140 g, 2.056 mmol) then phenyl glycidyl ether (0.200 g, 1.332 mmol). The reaction mixture was heated to 85 °C and maintained for 10 min. by microwave. The crude product was purified by column chromatography and provided α-(Phenoxymethyl)-1H-imidazole-1-ethanol (0.116 g, 55%) as white crystals. 1H-NMR (400 MHz, CHLOROFORM-D) δ 7.46 (s, 1H), 7.28-7.33 (m, 2H), 7.00 (t, J = 7.6 Hz, 1H), 6.93 (t, J = 8.0 Hz, 4H), 4.22-4.27 (m, 2H), 4.07-4.14 (m, 1H), 3.99 (q, J = 4.9 Hz, 1H), 3.92 (dd, J = 9.6 Hz, J = 6.0, 1H). 13C-NMR (101 MHz, CHLOROFORM-D) δ 158.18, 137.84, 129.74, 129.02, 121.61, 119.84, 114.57, 69.29, 68.72, 50.37.

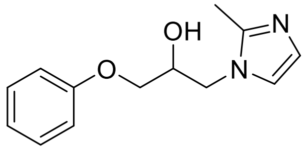

(3b) To a dry microwave tube was added 2-methylimidazole (0.164 g, 1.997 mmol) then phenyl glycidyl ether (0.200 g, 1.332 mmol). The reaction mixture was heated to 165 °C and maintained for 10 min. by microwave. The crude product was purified by column chromatography and provided 2-Methyl-α-(phenoxymethyl)-1H-imidazole-1-ethanol (.028, 53%) as off-white crystals. 1H-NMR (400 MHz, CHLOROFORM-D) δ 7.31 (m, 2H), 7.00 (t, 7.3 Hz, 1H), 6.85-6.93 (m, 4H), 4.25 (m, 1H), 4.17 (dd, J= 14.2 Hz, J = 4.6 Hz, 1H), 4.02 – 4.05 (m, 1H), 3.97 (d, J = 5 Hz, 2H), 2.39 (s, 3H). 13C-NMR (101 MHz, CHLOROFORM-D) δ 158.14, 145.20, 129.57, 126.51, 121.31, 119.84, 114.40, 69.04, 68.80, 49.41, 12.94

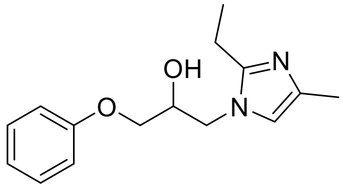

(3c) To a dry microwave tube was added 2-ethyl-4-methylimidazole (0.186 g, 1.688 mmol) then phenyl glycidyl ether (0.211g, 1.405 mmol). The reaction mixture was heated to 165 °C and maintained for 10 min. by microwave. The crude product was purified by column chromatography and provided 2-Ethyl-5-methyl-α-(phenoxymethyl)-1H-imidazole-1-ethanol (0.021 g, 49%) as white crystals. 1H-NMR (400 MHz, CHLOROFORM-D) δ 7.31 (m, 2H), 7.00 (m, 1H), 6.91 (m, 2H), 6.59 (s, 1H), 4.20-4.24 (m, 1H), 4.10 (dd, J = 14.4 Hz, J = 4.8 Hz, 1H), 3.93-4.01 (m, 3H), 2.68 (q, J = 8.2 Hz, 2H), 2.16 (s, 3H), 1.27-1.31 (m, 3H). 13C-NMR (101 MHz, CHLOROFORM-D) δ 158.20, 149.35, 135.88, 129.73, 121.58, 115.98, 114.56, 69.51, 68.91, 48.68, 20.08, 13.48, 12.65

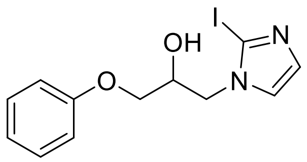

(3d) To a dry microwave tube was added 2-iodoimidazole (0.200 g, 1.031 mmol) then phenyl glycidyl ether (0.232 g, 1.545 mmol). The reaction mixture was heated to 60 °C and maintained for 10 min. by microwave. The product was precipitated from ethyl acetate and provided 2-Iodo-α-(phenoxymethyl)-1H-imidazole-1-ethanol (0.045 g, 21%) as white solid. 1H-NMR (400 MHz, CHLOROFORM-D) δ 7.30 (t, J = 7.1 Hz, 2H), 7.18 – 7.22 (m, 1H), 6.98-7.12 (m, 2H), 6.91 (d, J = 7.8 Hz, 2H), 4.20-4.30 (m, 2H), 4.02-4.13 (m, 2H), 3.90 (q, J = 4.7 Hz, 1H), 2.77 (s, 0H). 13C-NMR (101 MHz, CHLOROFORM-D) δ 157.93, 132.73, 129.66, 124.26, 121.65, 114.48, 90.30, 77.31, 77.00, 76.68, 69.32, 68.57, 51.70

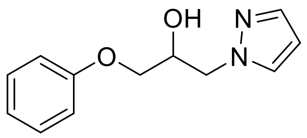

(3e) To a dry microwave tube was added pyrazole (0.2 g, 2.54 mmol) then phenyl glycidyl ether (0.190 g, 1.265 mmol). The reaction mixture was heated to 120 °C and maintained for 10 min. in the microwave. The crude product was purified by preparative TLC and provided 1-phenoxy-3-(1H-pyrazol-1-yl)propan-2-ol (.0619 g, 58%) as an off-white oil. 1H-NMR (400 MHz, CHLOROFORM-D) δ 7.25-7.29 (m, 2H), 6.87-6.98 (m, 3H), 5.79 (s, 1H), 4.80 (s, 1H), 4.16-4.33 (m, 3H), 4.01 (q, J = 4.6 Hz, 1H), 3.62 (t, J = 8.2 Hz, 1H), 2.21 (s, 3H), 2.18 (s, 3H). 13C-NMR (101 MHz, CHLOROFORM-D) δ 158.19, 148.20, 140.24, 129.55, 121.15, 114.38, 104.96, 69.35, 67.88, 49.29, 13.42, 10.81

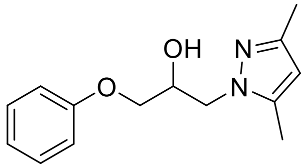

(3f) To a dry microwave tube was added 3,5-Dimethylpyrazole (0.1 g, 1.040 mmol) then phenyl glycidyl ether (0.190 g, 1.265 mmol). The reaction mixture was heated to 120 °C and maintained for 10 min. in the microwave. The crude product was purified by preparative TLC and provided 3,5-Dimethyl-α-(phenoxymethyl)-1H-pyrazole-1-ethanol (.0619 g, 55%) as an off-white oil. 1H-NMR (400 MHz, CHLOROFORM-D) δ 7.25-7.29 (m, 2H), 6.87-6.98 (m, 3H), 5.79 (s, 1H), 4.80 (s, 1H), 4.16-4.33 (m, 3H), 4.01 (q, J = 4.6 Hz, 1H), 3.62 (t, J = 8.2 Hz, 1H), 2.21 (s, 3H), 2.18 (s, 3H). 13C-NMR (101 MHz, CHLOROFORM-D) δ 158.19, 148.20, 140.24, 129.55, 121.15, 114.38, 104.96, 69.35, 67.88, 49.29, 13.42, 10.81

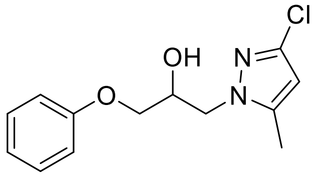

(3g) To a dry microwave tube was added 3-chloro-5-methyl-1H-pyrazole (0.200 g, 1.716 mmol) then phenyl glycidyl ether (0.515 g, 3.429 mmol). The reaction mixture was heated to 120 °C and maintained for 5 min. by microwave. The crude product was purified by preparative TLC and provided 3-Chloro-5-methyl-α-(phenoxymethyl)-1H-pyrazole-1-ethanol (.117 g, 26%) as a clear oil. 1H-NMR (400 MHz, CHLOROFORM-D) δ 7.28 (dd, J = 8.6 Hz, J = 7.4 Hz, 2H), 6.88-6.98 (m, 3H), 6.00 (s, 1H), 4.24-4.39 (m, 3H), 4.01-4.11 (m, 2H), 3.82 (dd, J = 9.6 Hz, J = 6.4 Hz, 1H), 2.22 (s, 3H). 13C-NMR (101 MHz, CHLOROFORM-D) δ 158.24, 149.27, 129.51, 128.16, 121.22, 114.49, 104.26, 69.25, 68.35, 50.04, 13.97

Figure S1. 1H NMR spectra of compound 3a.

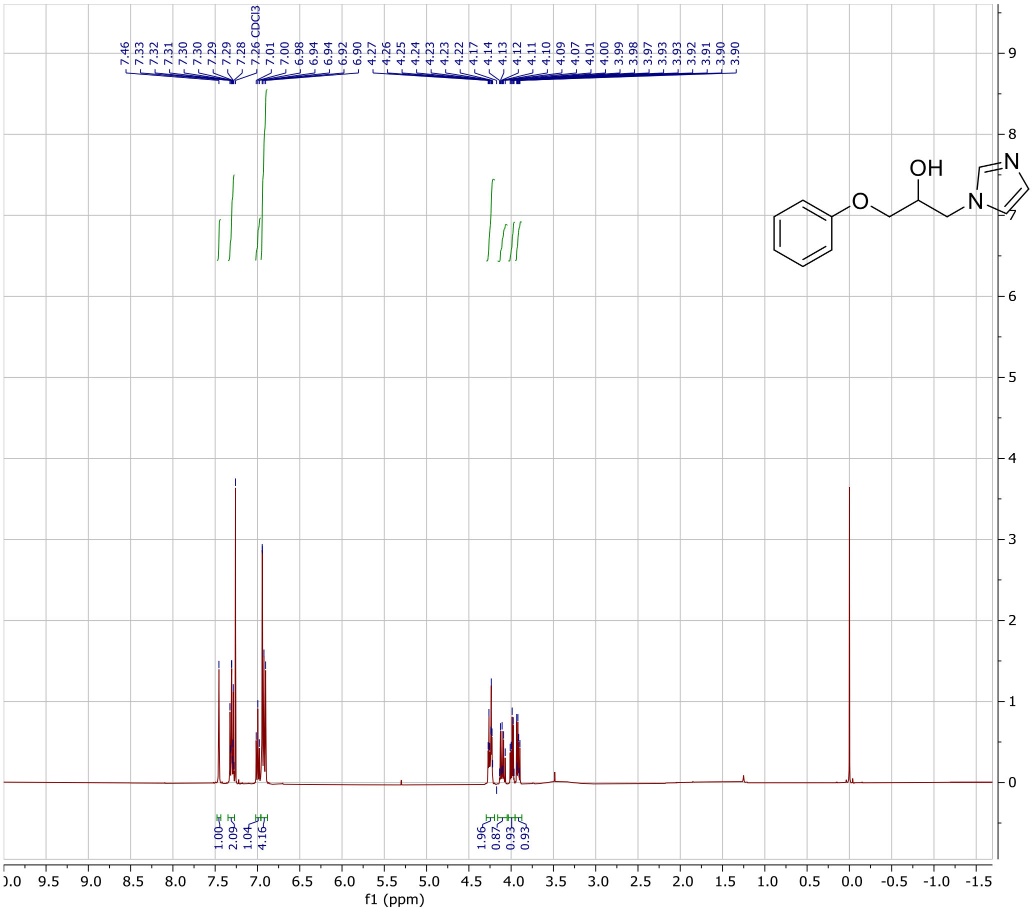

Figure S2. 13C NMR spectra of compound 3a.

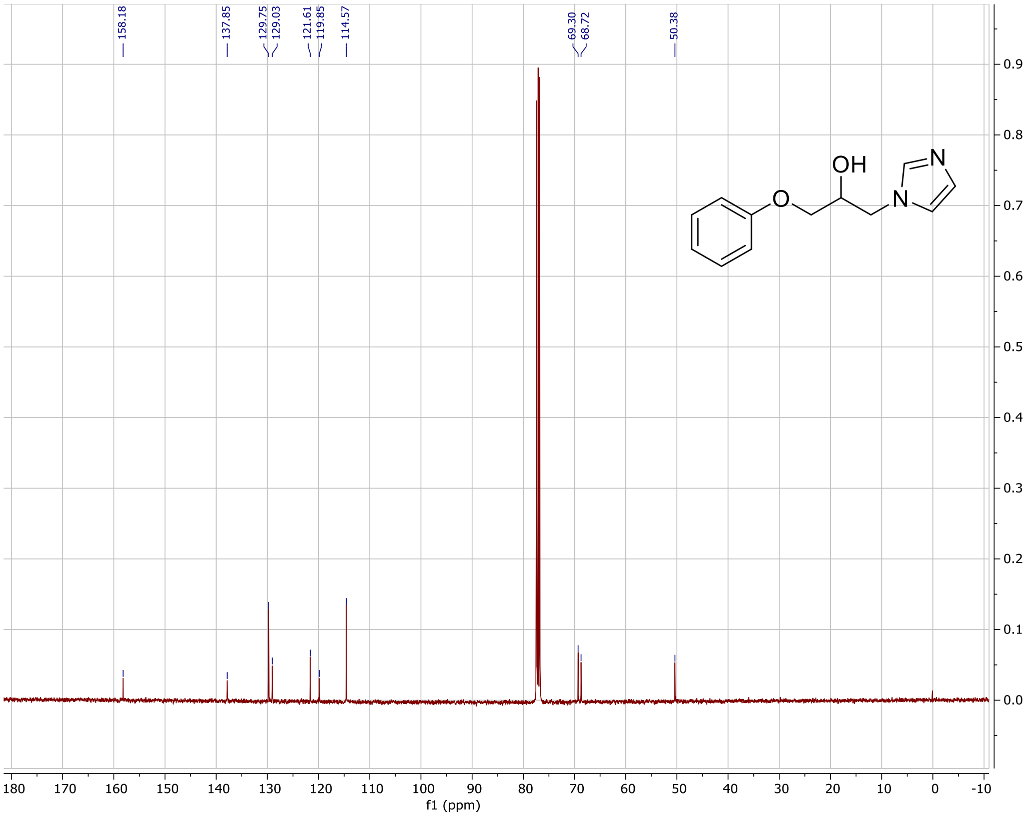

Figure S3. 1H NMR spectra of compound 3b.

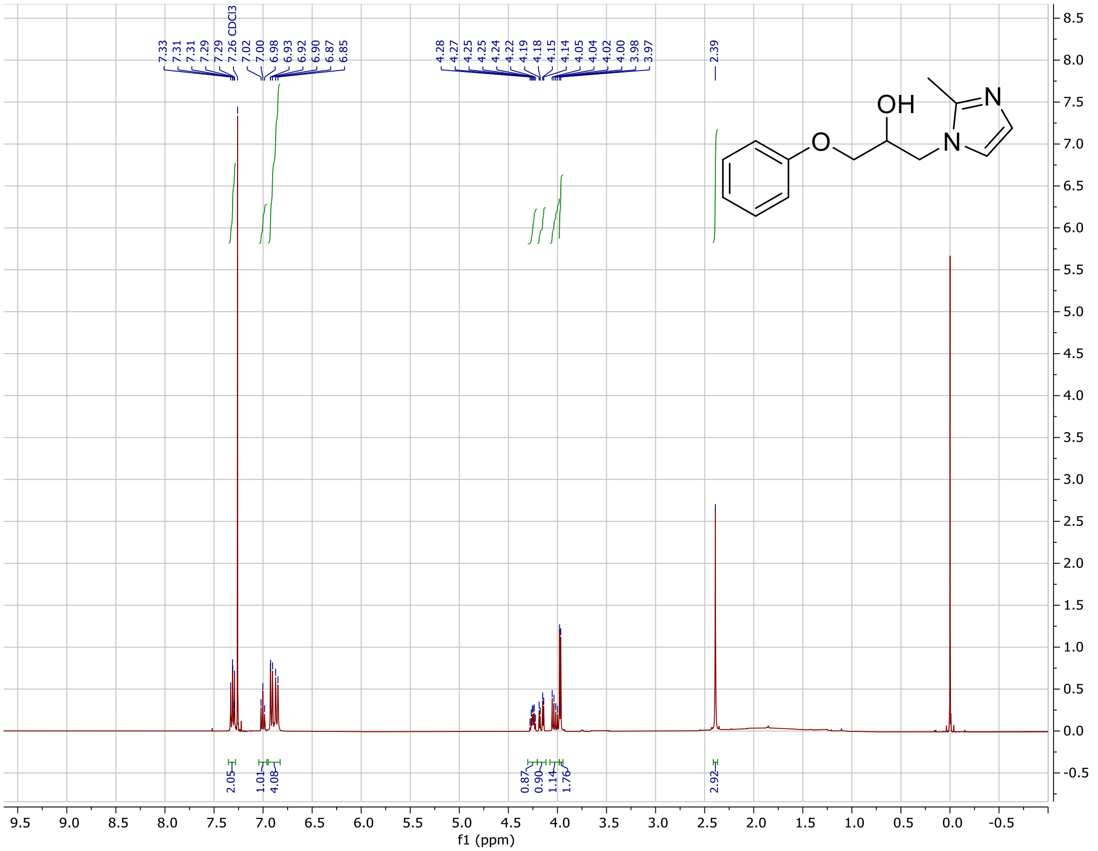

Figure S4. 13C NMR spectra of compound 3b.

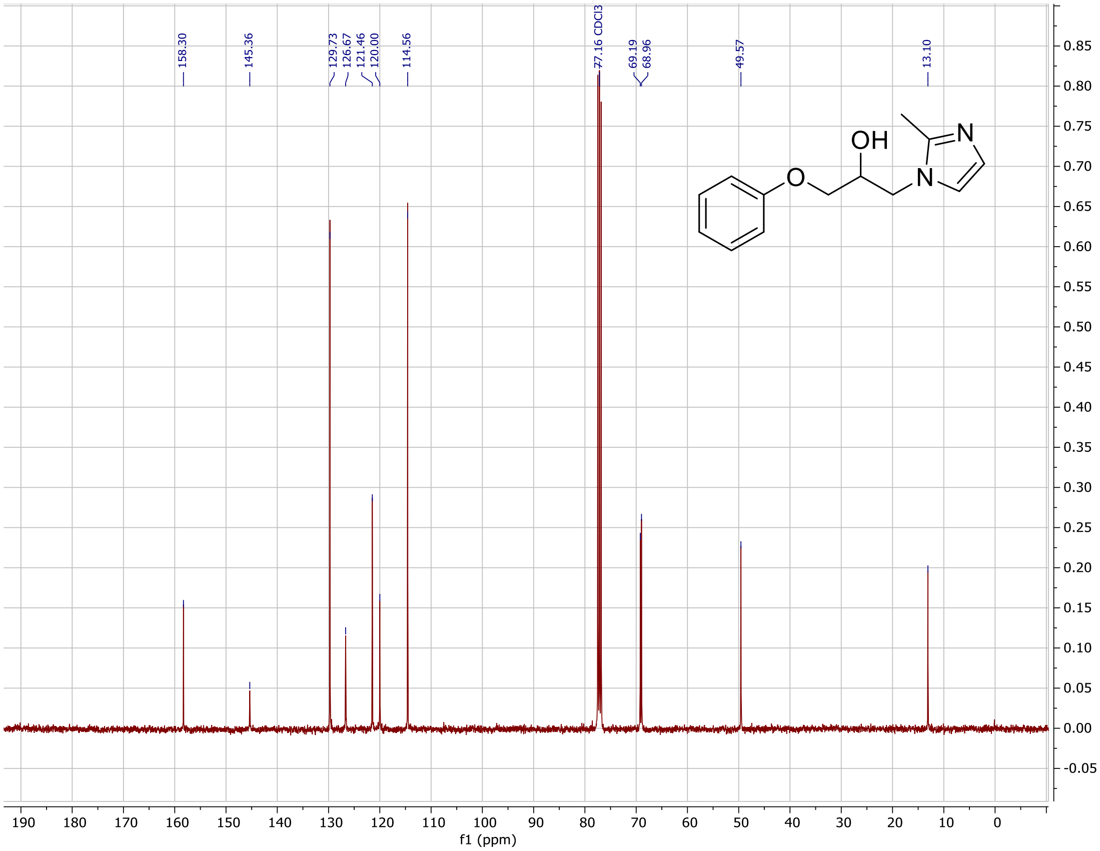

Figure S5. 1H NMR spectra of compound 3c.

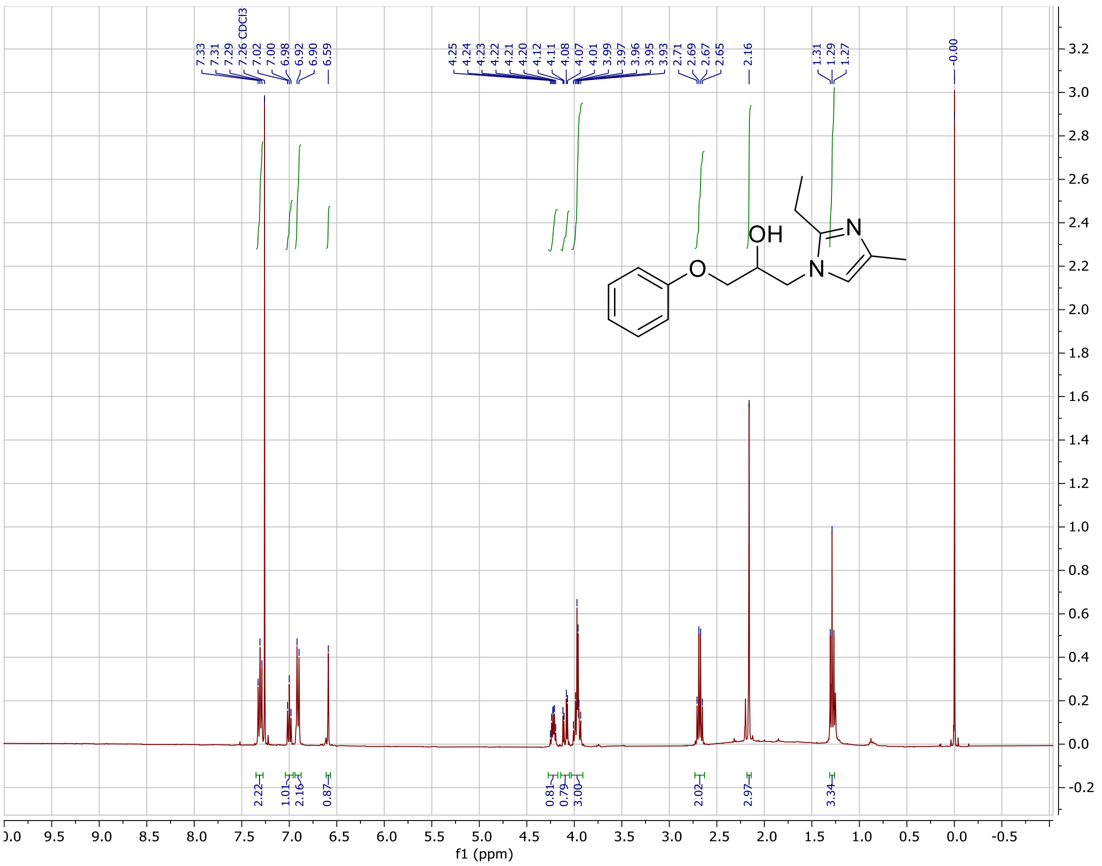

Figure S6. 13C NMR spectra of compound 3c.

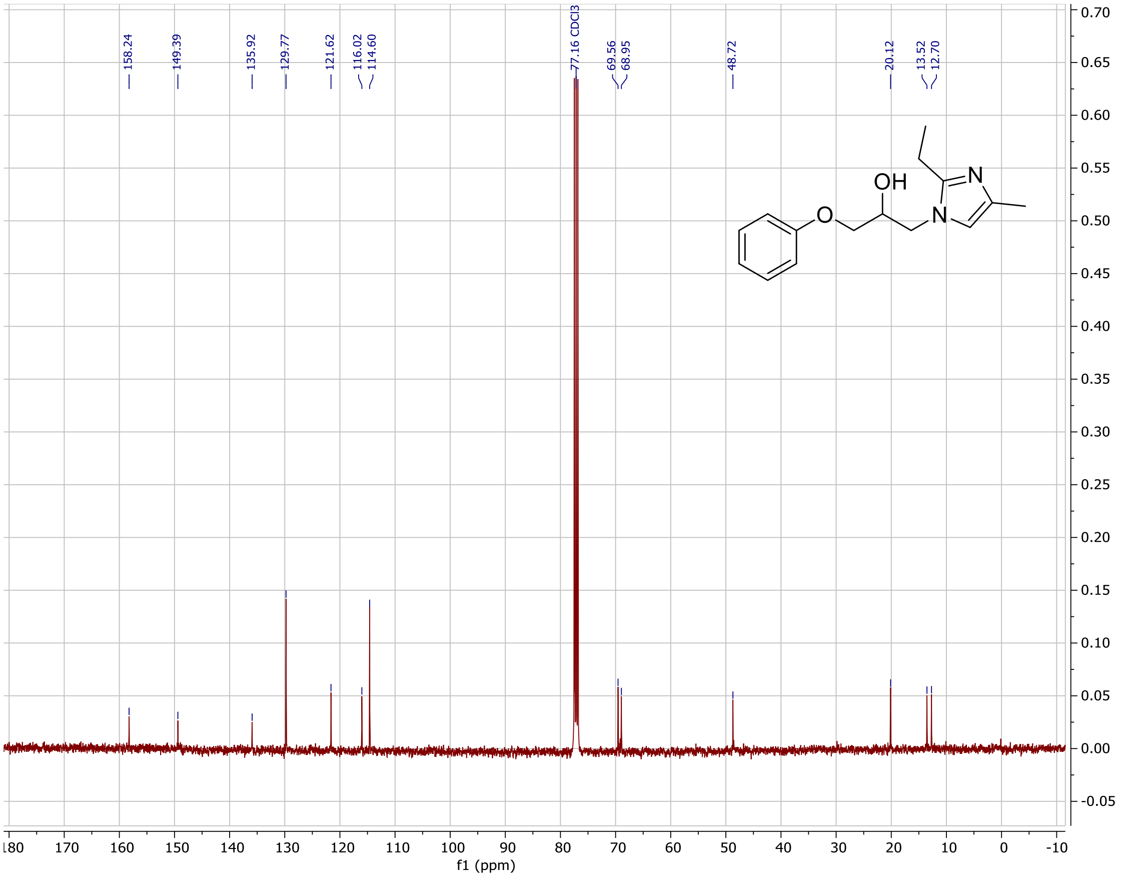

Figure S7. 1H NMR spectra of compound 3d.

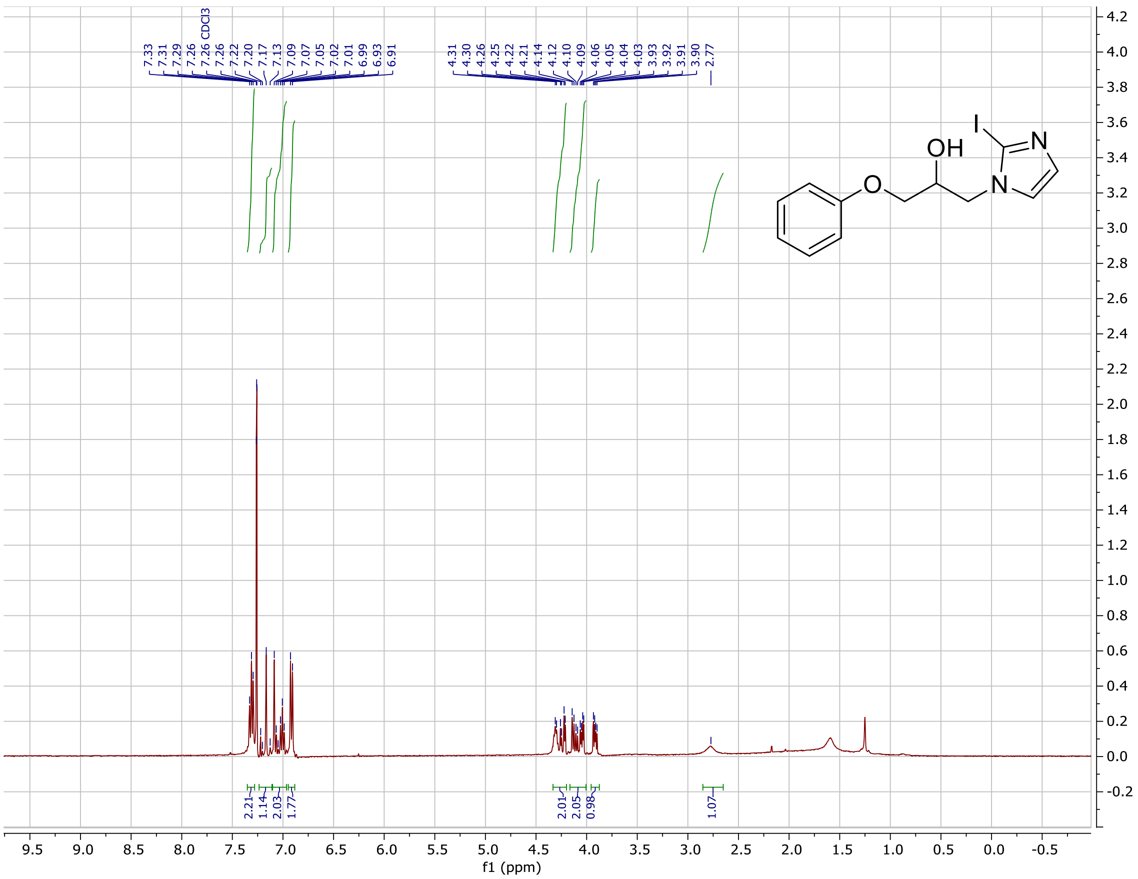

Figure S8. 13C NMR spectra of compound 3d.

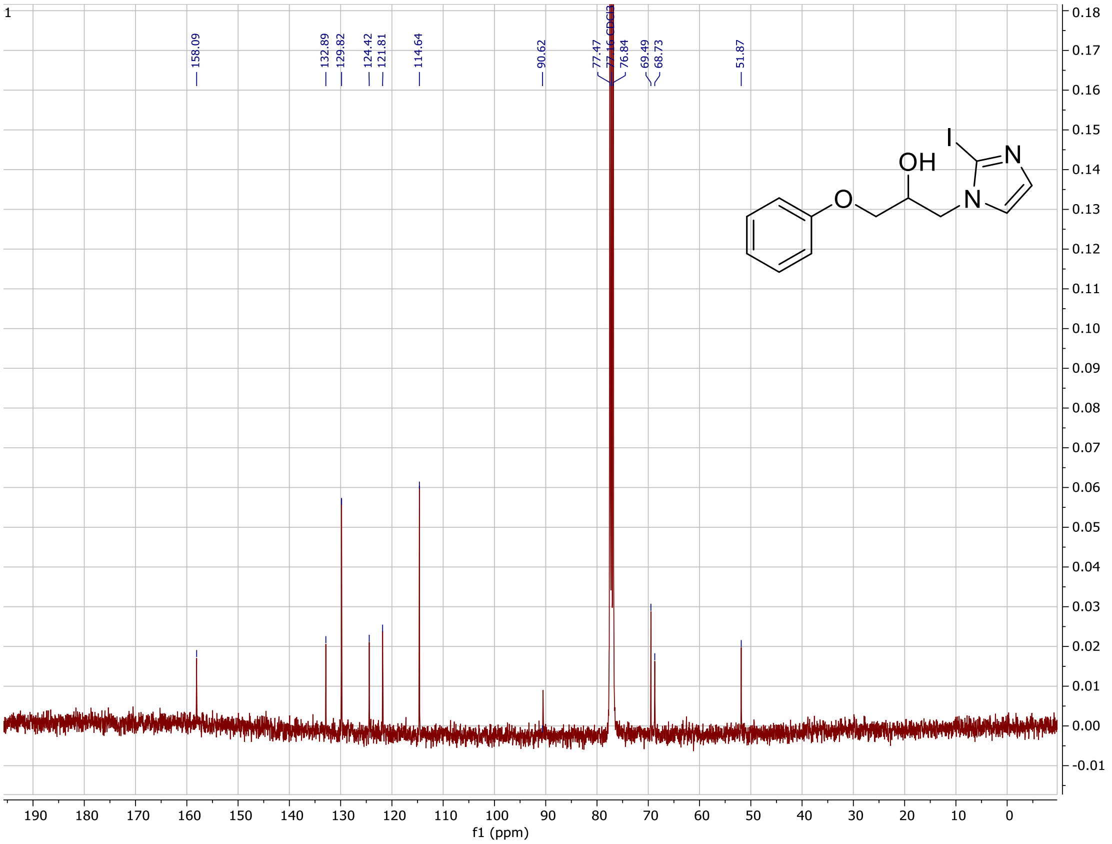

Figure S9. 1H NMR spectra of compound 3e.

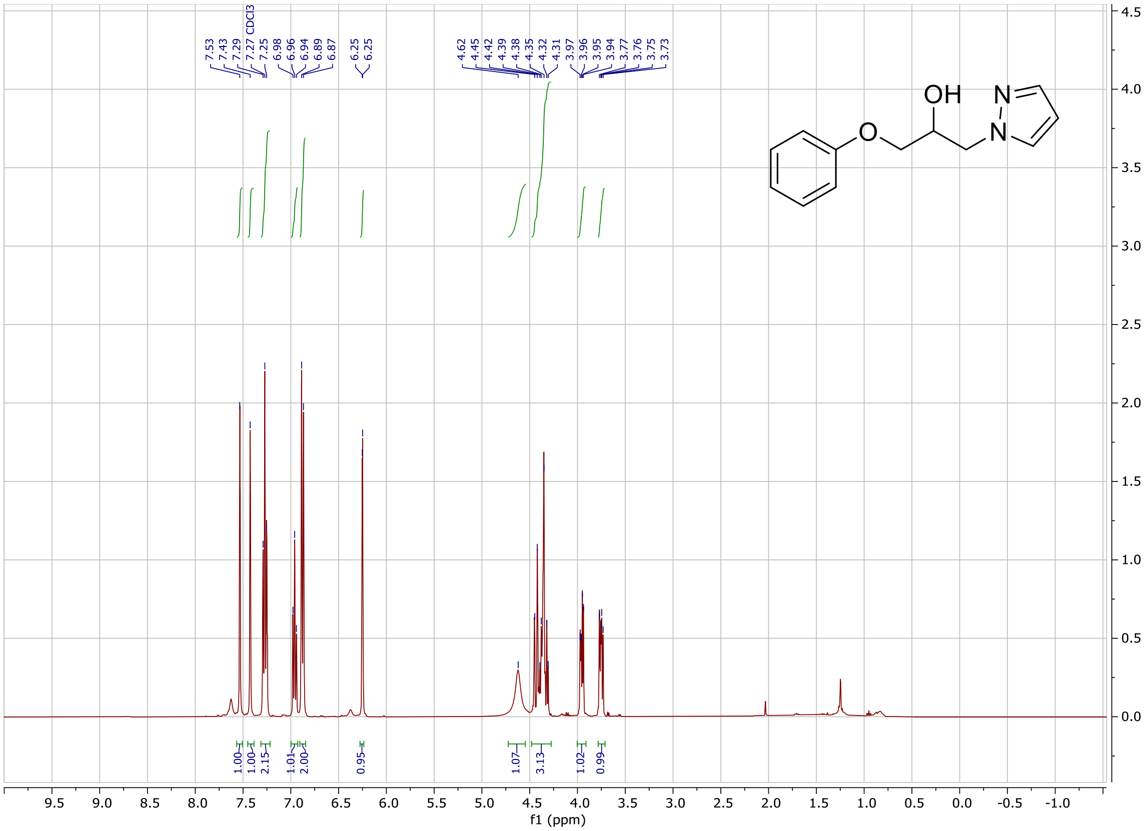

Figure S10. 13C NMR spectra of compound 3e.

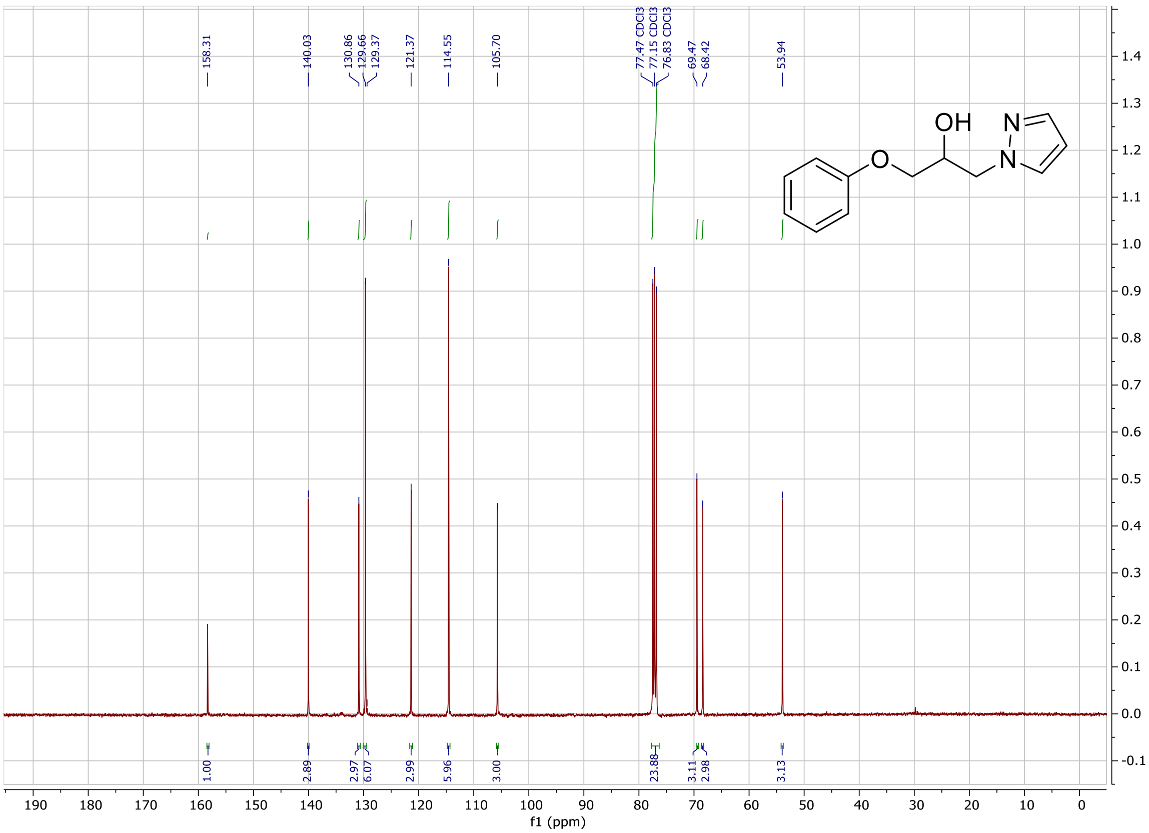

Figure S11. 1H NMR spectra of compound 3f.

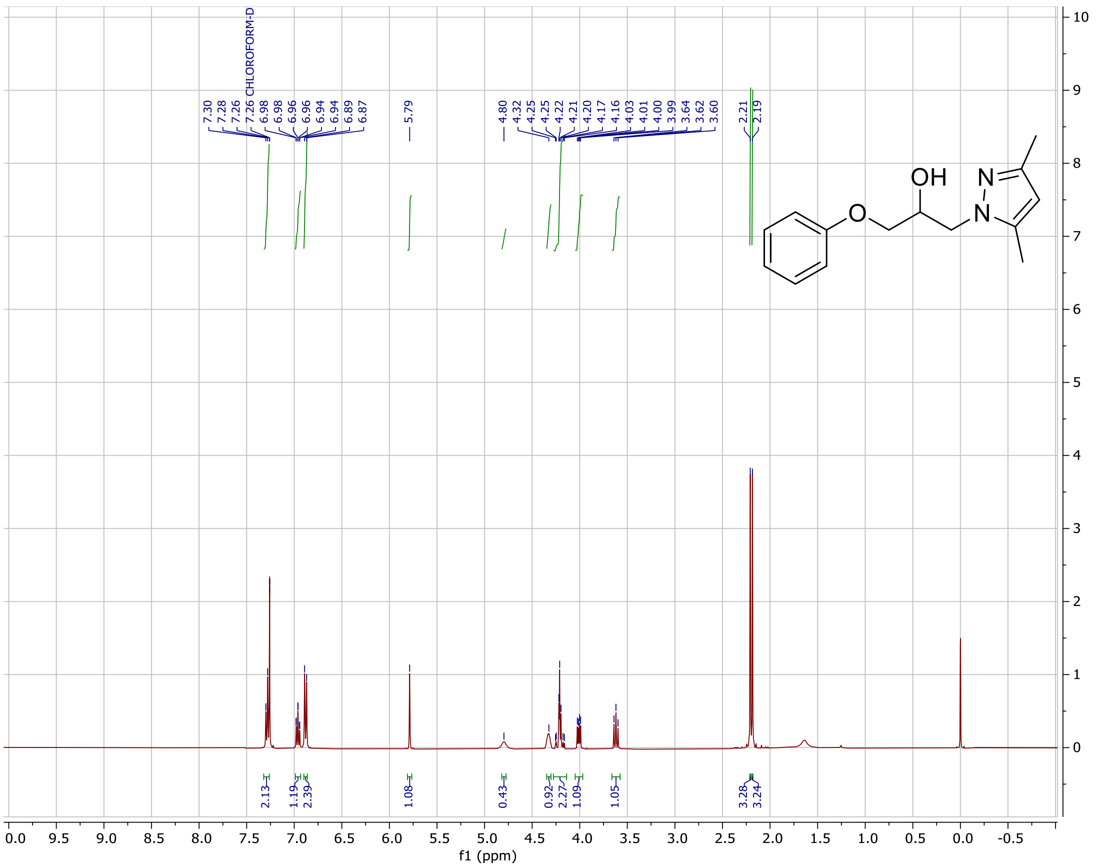

Figure S12. 13C NMR spectra of compound 3f.

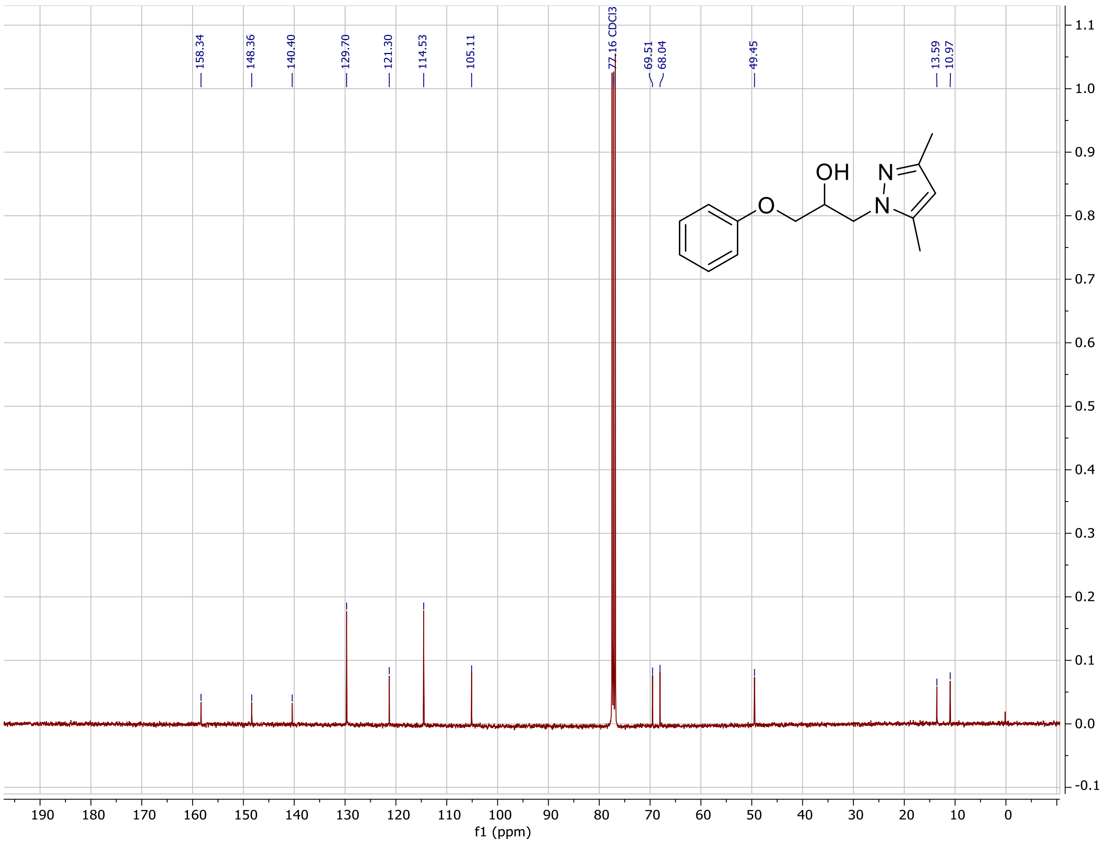

Figure S13. 1H NMR spectra of compound 3g.

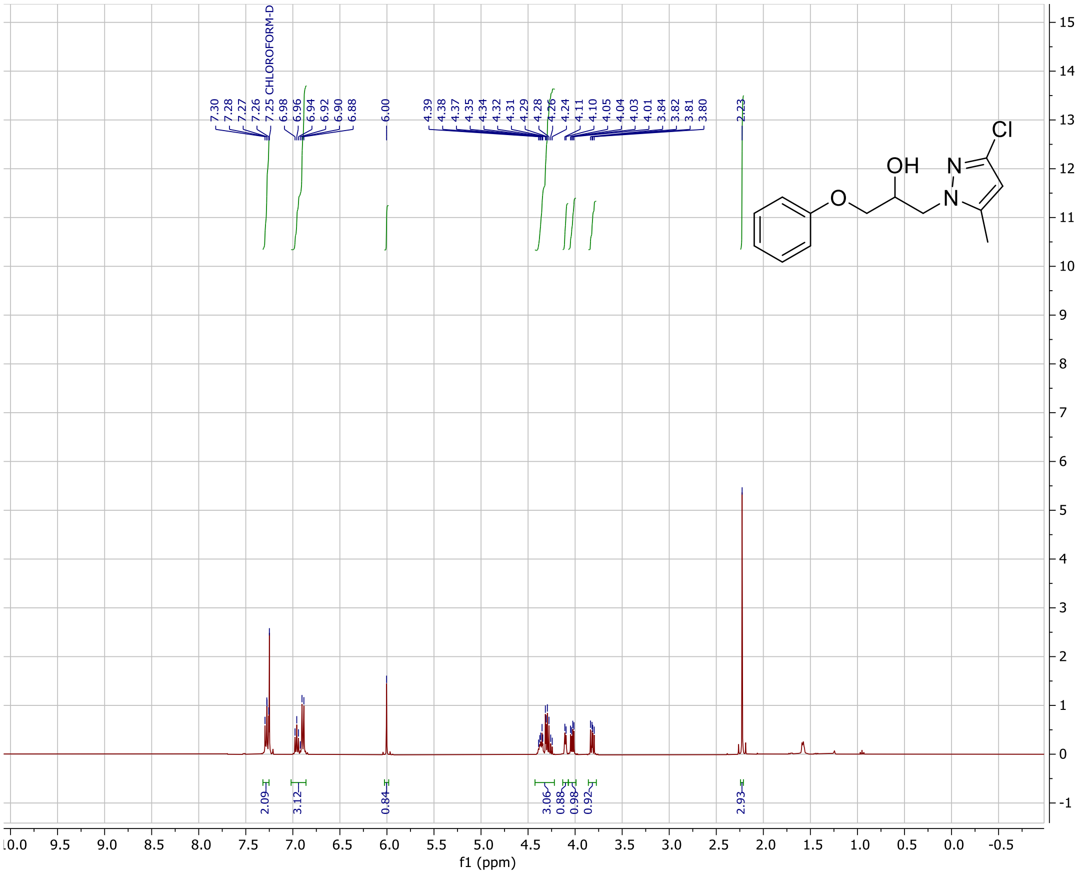

Figure S14. 13C NMR spectra of compound 3g.

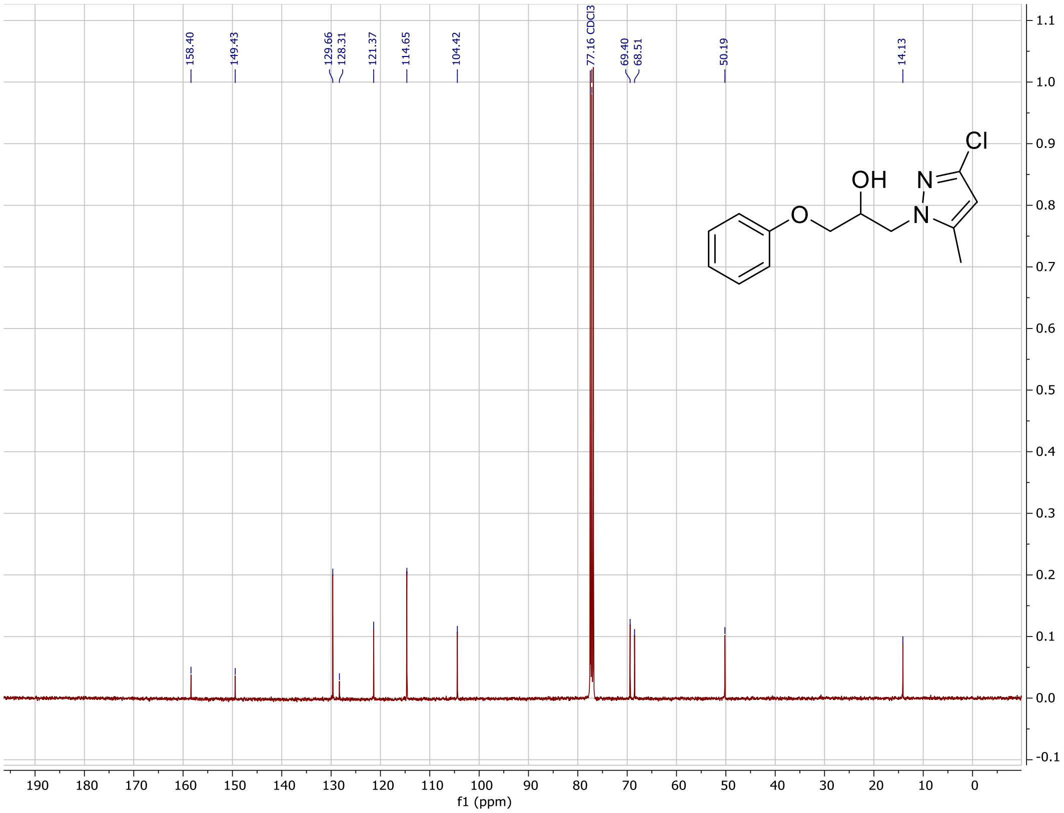

Supplement: Supplementary file 1 [file molecules-30-01760-s001.zip › molecules-3480046-supplementary.pdf]
